# GSK3 is required for rapalogs to induce degradation of some oncogenic proteins and to suppress cancer cell growth

**DOI:** 10.18632/oncotarget.3291

**Published:** 2015-03-12

**Authors:** Junghui Koo, Xuerong Wang, Taofeek K. Owonikoko, Suresh S. Ramalingam, Fadlo R. Khuri, Shi-Yong Sun

**Affiliations:** ^1^ Department of Hematology and Medical Oncology, Emory University School of Medicine and Winship Cancer Institute, Atlanta, GA, USA; ^2^ Department of Pharmacology, Nanjing Medical University, Nanjing, Jiangsu, China

**Keywords:** GSK3, mTOR, rapamycin, degradation, oncogenic proteins

## Abstract

The single-agent activity of rapalogs (rapamycin and its analogues) in most tumor types has been modest at best. The underlying mechanisms are largely unclear. In this report, we have uncovered a critical role of GSK3 in regulating degradation of some oncogenic proteins induced by rapalogs and cell sensitivity to rapalogs. The basal level of GSK3 activity was positively correlated with cell sensitivity of lung cancer cell lines to rapalogs. GSK3 inhibition antagonized rapamycin's growth inhibitory effects both *in vitro* and *in vivo*, while enforced activation of GSK3β sensitized cells to rapamycin. GSK3 inhibition rescued rapamcyin-induced reduction of several oncogenic proteins such as cyclin D1, Mcl-1 and c-Myc, without interfering with the ability of rapamycin to suppress mTORC1 signaling and cap binding. Interestingly, rapamycin induces proteasomal degradation of these oncogenic proteins, as evidenced by their decreased stabilities induced by rapamcyin and rescue of their reduction by proteasomal inhibition. Moreover, acute or short-time rapamycin treatment dissociated not only raptor, but also rictor from mTOR in several tested cell lines, suggesting inhibition of both mTORC1 and mTORC2. Thus, induction of GSK3-dependent degradation of these oncogenic proteins is likely secondary to mTORC2 inhibition; this effect should be critical for rapamycin to exert its anticancer activity.

## INTRODUCTION

The mammalian target of rapamycin (mTOR) is a serine-threonine protein kinase belonging to the family of the phosphatidylinositol 3-kinase-related kinases [1]. It critically regulates various biological functions, particularly cell growth, metabolism and survival, largely through forming two distinct complexes with different partner proteins: raptor (mTOR complex 1; mTORC1) and rictor (mTOR complex 2; mTORC2) [2–4]. The mTORC1 signaling primarily regulates cap-dependent protein translation initiation, an essential process for synthesis of many oncogenic proteins such as cyclin D1, c-Myc, Mcl-1, HIF1α and VEGF [3, 4]. This signaling pathway is often dysregulated (activated) in various types of human cancers, largely due to hyper-activation of PI3K/Akt and Ras/Raf/MEK/ERK signaling, and hence has emerged as an attractive cancer therapeutic target [5–7]. The conventional mTOR inhibitors, rapamycin and its analogues (rapalogs), are specific allosteric inhibitors of mTOR. It is generally thought that these rapalogs have weak activity against the mTORC2 while strongly inhibiting mTORC1 activity. In the clinic, the single-agent activity of rapalogs in most tumor types has been modest at best although some of them (e.g., everolimus) have been approved by the FDA for treatment of several types of cancers such as advanced renal cell cancer and pancreatic neuroendocrine tumors [8]. Currently, it is unclear why most types of cancers respond poorly to rapalog monotherapy.

Glycogen synthase kinase-3 (GSK3) is a ubiquitous serine/threonine kinase with two isoforms: α and β, in mammals, encoded by different genes [9, 10]. GSK3 regulates many cellular functions such as glycogen metabolism, insulin signaling, cell proliferation, apoptosis and neuronal function [9, 10]. Thus, GSK3 inhibition has been considered an attractive therapeutic strategy for certain human diseases such as diabetes, neurodegenerative diseases, muscle hypertrophy, and mental disorders [11, 12]. Accordingly, several potent GSK3 inhibitors have been developed, most of which are ATP competitive and inhibit both GSK3α and GSK3β [12]. GSK3 has also been implicated in regulation of oncogenesis, but with complex patterns: it acts paradoxically as a tumor suppressor in some cancers while potentiating growth in others [13–15]. Because of the concern that inhibition of GSK3 may promote oncogenesis, the progress of GSK3 inhibitors into clinical trials has been clouded [15].

Our recent study has demonstrated that maintaining GSK3 activity is critical for mTOR kinase inhibitors to exert their anticancer activity [16]. The current study has focused on determining whether GSK3 activity also impacts the anticancer efficacies of rapalogs and on understanding the underlying mechanisms. Out results have shown that GSK3 is also an important determinant for cancer cells to respond to rapalogs. In addition to cyclin D1, rapalogs induce GSK3-dependent degradation of other oncogenic proteins such as c-Myc and Mcl-1; these effects should be tightly associated with rapalogs’ cancer therapeutic efficacies.

## RESULTS

### Pharmacological inhibition of GSK3 antagonizes rapamycin's effects on inhibiting the growth of NSCLC cells *in vitro* and *in vivo*

**Figure 1:**
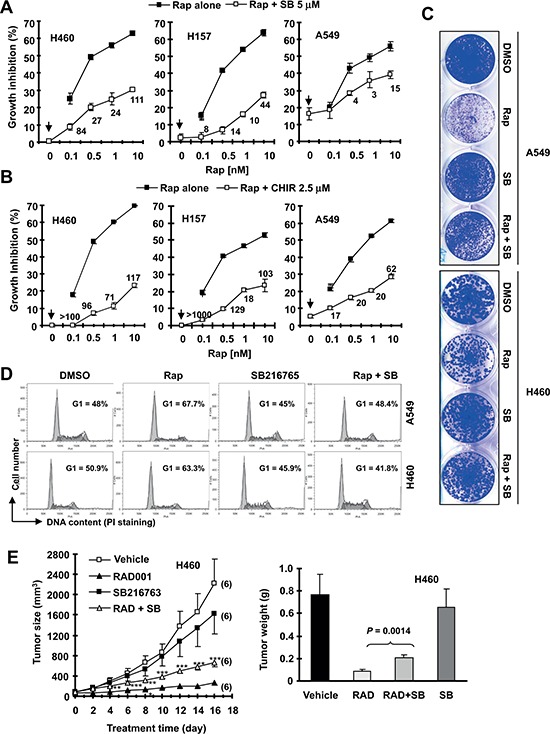
The presence of the GSK3 inhibitor, SB216763 or CHIR99021, antagonizes rapamycin's growth inhibitory effects evaluated in a 3-day monolayer culture assay (A and B), in an 8-day colony formation assay (C), by cell cycle analysis (D) and in a nude mice xenograph model (E) **(A and B)**, The given lung cancer cell lines were plated on 96-well cell culture plates and treated next day with the indicated concentrations of rapamycin (Rap) alone, SB216763 (SB) or CHIR99021 (CHIR) alone (as indicated by arrow inside the graphs) or their combination. After 3 days, cell numbers were estimated using the SRB assay and CIs were calculated with CompuSyn software and labeled inside the graphs. Data, means of four replicated determinations; Bars, ± SDs. **(C)**, The indicated cell lines at a density of approximately 400 cells/well were seeded in 12-well plates. On the second day, the cells were treated with DMSO, 10 nM rapamycin, 5 μM SB216763, or rapamycin plus SB216763 (SB). After 8 days, the plates were stained for the formation of cell colonies with crystal violet dye and pictured using a digital camera. **(D)**, The indicated lung cancer cell lines were treated with DMSO, 10 nM rapamycin, 5 μM SB216763, or rapamycin plus SB216763 for 48 h and then harvested for cell cycle analysis by flow cytometry. **(E)**, H460 xenografts were treated with indicated agents. This study was done in conjunction with our previous INK128 and SB216763 combination experiment and thus the vehicle and SB216763 groups were shared to minimally use mice (see details in “Materials and Methods”). Tumor sizes were measured once every two days. Each measurement is a mean ± SE (*n* = 6). At the end of the experiment, tumor xenografts were removed and weighed. **P < 0.01; ***P < 0.001 compared with RAD001 group alone using the student *t* test.

In three rapamycin-sensitive human non-small cell lung carcinoma (NSCLC) cell lines H460, A549 and H157, we found that the combination of rapamycin with the GSK3 inhibitor SB216763 or CHIR99021 resulted in attenuation of growth suppression of these NSCLC cell lines (Figs. 1A and 1B). The combination indexes (CIs) in every tested cell line were far greater than 1, indicating super-antagonistic effects. Similar antagonistic effects were also observed in other NSCLC cell lines (e.g., EKVX, H226 and H292; Supplementary Fig. S1). In a long-term colony formation assay that allows us to repeat the treatments, the combination of SB216763 and rapamycin was also less effective than rapamycin alone in inhibiting colony growth of both A549 and H460 cell lines (Fig. 1C), further confirming the antagonistic effects. Furthermore, rapamycin induced G1 arrest in both A549 and H460 cells; however this effect was abrogated by the addition of SB216763 (Fig. 1D). Collectively, these results robustly indicate that inhibition of GSK3 impairs the ability of rapamycin to suppress the growth of NSCLC cells.

We further conducted lung cancer xenograft experiment in nude mice to demonstrate whether GSK3 activity is important for the anticancer activity of rapalogs *in vivo*. RAD001 alone very effectively inhibited the growth of H460 xenografts; however, the combination of RAD001 with SB216763 was significantly weaker than RAD001 alone in inhibiting the growth of the xenografts (Fig. 1E) although it did not apparently affect the body weights of mice (data not shown). This result was generated in conjunction with our previous INK128 and SB216763 combination experiment as described in our previous study [16] and thus the vehicle and SB216763 groups were shared in order to minimally use mice. Hence, it is clear that inhibition of GSK3 attenuates the ability of RAD001 to inhibit the growth of lung cancer xenografts in nude mice, indicating that GSK3 activity is also critical for rapalogs’ antitumor activity *in vivo*.

### Genetic manipulation of GSK3 expression alters cell response to rapalogs

**Figure 2:**
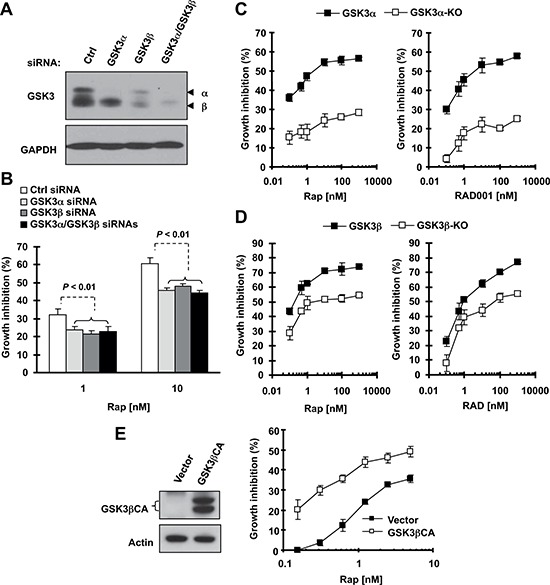
Knockdown (A and B) or knockout of GSK3 (C and D) and enforced expression of constitutively activated form of GSK3β (E) modulate cell responses to rapalogs **(A and B)** A549 cells were transfected with control (Ctrl), GSK3α, GSK3β or GSK3α plus GSK3β siRNAs for 24 h and then re-seeded in 96-well plates. After 48 h, the cells were exposed to 1 or 10 nM rapamycin (Rap) for an additional 3 days. Cell numbers were estimated with the SRB assay (B). GSK3 knockdown effects at 48 h were detected with Western blotting (A). **(C and D)** The indicated MEFs were seeded in 96-well plates and next day treated with different concentrations of rapamycin or RAD001 as indicated. After 3 days, cell numbers were estimated with the SRB assay. **(E and F)** H1299 cells were transfected with empty vector or expression plasmid carrying GSK3βCA and re-seeded in 96-well plates 24 h later. After 48 h, the cells were exposed to different concentrations of rapamycin as indicated for an additional 3 days. Cell numbers were estimated with the SRB assay (right panel). GSK3βCA expression was confirmed with Western blotting (left panel). Data, means of four replicated determinations; Bars, ± SDs.

To further validate our finding, we examined the impact of specific genetic inhibition of GSK3, including gene knockdown and knockout, on cell responses to a rapalog. Small interfering RNA (siRNA)-mediated knockdown of GSK3α, GSK3β or both were confirmed with Western blotting (Fig. 2A). In a 3-day growth-inhibition assay, knockdown of GSK3α, GSK3β or both significantly reduced cell sensitivity to rapamycin (Fig. 2B). Consistently, GSK3α-KO or GSK3β-KO murine embryonic fibroblasts (MEFs) were drastically less sensitive to both rapamycin and RAD001 in comparison with wild-type (WT) MEFs (Figs. 2C and 2D). Taken together, these data further support the notion that inhibition of GSK3 impairs cell response to rapalogs.

Furthermore, we asked whether GSK3 activation can increase cell sensitivity to rapamycin. To this end, we transfected a constitutively active form of GSK3β (GSK3βCA) into H1299 cells (this cell line has a high transfection efficiency and relatively low sensitivity to rapalogs) and then analyzed its impact on cell response to rapamycin. Compared with vector control-transfected cells, GSK3βCA-transfected cells were much more sensitive to rapamycin treatment (Fig. 2E). Thus, it is clear that increased GSK3 activation, in contrast to GSK3 inhibition, sensitizes cells to rapamycin.

### Basal level of GSK3 activity in NSCLC cell lines is significantly correlated with cell sensitivity to rapalogs

**Figure 3:**
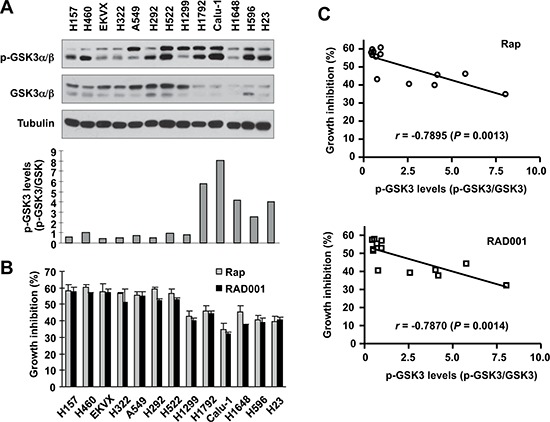
Basal levels of p-GSK3 in human lung cancer cell lines (A) are inversely correlated with cell sensitivity to rapalogs (B and C) Whole-cell lysates were prepared from the listed cell lines with comparable cell densities and subjected to Western blotting for detection of the indicated proteins **(A)**. The intensities of these proteins were quantified with NIH Image J software. The growth-inhibitory effects of rapamycin or RAD001 at 10 nM were determined with the SRB assay after 3 days. The correlation between p-GSK3/GSK3 and growth inhibition was calculated with GraphPad InStat software **(B and C)**.

Following these findings, we further determined whether basal levels of GSK3 activity are associated with cell sensitivity to rapalogs. Here we detected basal levels of p-GSK3 as an indication of inactivated or low GSK3 activity in a panel of 13 NSCLC cell lines by Western blotting (Fig. 3A). These cell lines possessed different sensitivities to rapamycin or RAD001 as determined in a 3-day growth-inhibitory assay (Fig. 3B). Correlation analysis showed that high p-GSK3 levels (i.e., low GSK3 activity) were significantly associated with weak growth-inhibitory effect of rapamycin (*r* = 0.7895, *P* = 0.0013) or RAD001 (*r* = 0.7870, *P* = 0.0014) (Fig. 3C), meaning that low GSK3 activity is associated with reduced cell sensitivity to rapalogs (e.g., in NSCLC cell lines).

### Inhibition of GSK3 does not interfere with the ability of rapamycin to inhibit mTORC1 signaling and cap binding, but blocks rapamycin-induced reduction of cyclin D1, c-Myc and Mcl-1

**Figure 4:**
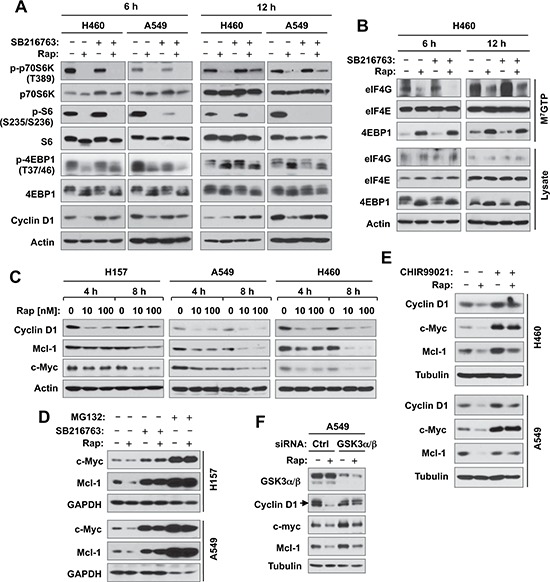
Inhibition of GSK3 with SB216763 or siRNA rescues rapamycin-induced reduction of cyclin D1, c-Myc and Mcl-1 (A, C-F) without blocking rapamycin-mediated suppressive effects on mTORC1 signaling (A) and on cap binding (B) **(A and B)**, The indicated cell lines were treated with DMSO, 10 nM rapamycin (Rap), 5 μM SB216763, or rapamycin plus SB216763 for 6 h or 12 h. **(C)**, The indicated cell lines were exposed to different concentrations of rapamycin for 4 or 8 h. **(D)**, The indicated cell lines were treated with DMSO, 10 nM rapamycin, 5 μM SB216763, 10 μM MG132, rapamycin plus SB216763, or rapamycin plus MG132 for 6 h. **(E)**, The indicated cell lines were treated with DMSO, 10 nM rapamycin, 10 μM CHIR99021, or rapamycin plus CHIR99021 for 6 h. **(F)**, A549 cells were transfected with the given siRNAs and after 48 h were exposed to 10 nM rapamycin for 6 h. After the aforementioned treatments, the cells were then harvested for preparation of whole-cell protein lysates and subsequent Western blotting. Moreover, lysates from H460 cells (B) were also used to the m^7^GTP pull-down and subsequent detection of the given proteins by Western blot analysis (B).

To understand the mechanism by which GSK3 activity regulates cell response to rapalogs, we then determined whether GSK3 inhibition interferes with the ability of rapamycin to inhibit the mTORC1 signaling and cap-dependent translation given the general thought that rapamycin primarily inhibits mTORC1. In two tested NSCLC cell lines, H460 and A549, rapamycin at 6 h treatment was equally effective in decreasing the levels of p-p70S6K, p-4EBP1 and p-S6, which are well-known readouts of the mTORC1, both in the absence and presence of SB216763. At 12 h treatment, the presence of SB216763 slightly rescued the reduction of p-pS70SK by rapamycin, but did not prevent rapamycin-induced decrease of either p-S6 or p-4EBP1 (Fig. 4A). These results together indicate that inhibition of GSK3 does not interfere with the ability of rapamycin to inhibit the mTORC1 signaling. Furthermore we compared the effects of rapamycin with and without SB216763 on cap-binding of the eIF4F complex. In this experiment, rapamycin effectively reduced the amounts of eIF4G bound to eIF4E with increased amounts of 4EBP1 bound to eIF4E regardless of the presence or absence of SB216763 (Fig. 4B), suggesting that inhibition of GSK3 does not impair the ability of rapamycin to suppress cap-dependent translation initiation either. Under the same conditions, rapamycin decreased the levels of cyclin D1, an oncogenic protein known to be regulated by mTORC1-mediated cap-dependent translation. Interestingly, co-treatment of the cells with SB216763 and rapamycin prevented cyclin D1 reduction induced by rapamcyin in both tested cell lines (Fig. 4A).

In addition to translation regulation, cyclin D1 is known to be regulated at the posttranslational level through GSK3-dependent protein degradation [17, 18]. Hence, we examined other two proteins, c-Myc and Mcl-1, known to be regulated by both cap-dependent translation and GSK3-dependent protein degradation mechanisms [5, 19, 20]. Like cyclin D1, rapamycin reduced the levels of both c-Myc and Mcl-1 in 3 tested NSCLC cell lines even early at 4 h post treatment (Fig. 4C). The presence of SB216763 rescued the reduction of both c-Myc and Mcl-1 induced by rapamycin (Fig. 4D). Moreover, we tested the effects of another GSK3 inhibitor, CHIR99021, and GSK3 knockdown on rapamycin-induced reduction of cyclin D1, c-Myc and Mcl-1. In agreement with the findings using SB216763, both CHIR99021 (Fig. 4E) and GSK3 knockdown (Fig. 4F) rescued reduction of these proteins induced by rapamycin. Thus, it is clear that rapamycin induces a GSK3-dependent reduction of cyclin D1, c-Myc and Mcl-1, likely independent of translation regulation.

### Rapamycin decreases the levels of cyclin D1, c-Myc and Mcl-1 through promoting their degradation

**Figure 5:**
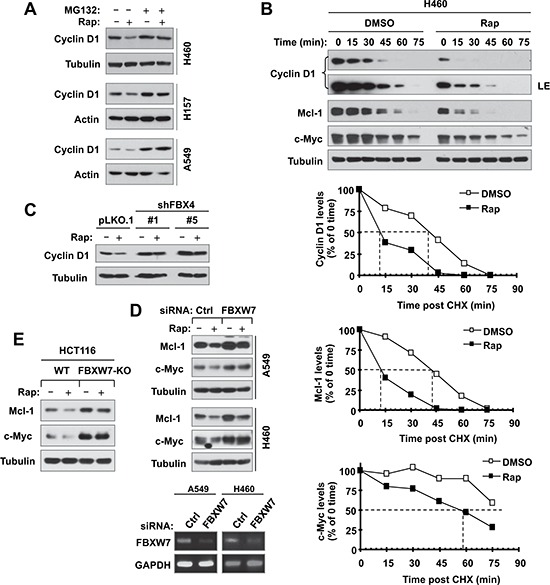
Rapamycin decreases the levels of cyclin D1, c-Myc and Mcl-1 through facilitating their degradation (A and B), which is mediated by either FBX4 (C) or FBXW7 (D and E) **(A)**, The indicated cell lines were pre-treated with 10 μM MG132 for 30 min and then co-treated with 10 nM rapamycin for 6 h. **(B)**, H460 cells were treated with DMSO or 10 nM of rapamycin for 6 h. The cells were then washed with PBS 3 times and re-fed with fresh medium containing 10 μg/ml CHX. At the indicated times, the cells were harvested for preparation of whole-cell protein lysates and subsequent Western blot analysis. Protein levels were quantified with NIH Image J Software and were normalized to tubulin. **(C)** The indicated A549 transfectants were exposed to 10 nM rapamycin for 6 h. **(D)** The indicated cells were transfected with the given siRNAs and after 48 h were exposed to 10 nM rapamycin for an additional 6 h. **(E)** The indicated cell lines were exposed to 10 nM rapamycin for 6 h. After the aforementioned treatments (A, C–E), the cells were then harvested for preparation of whole-cell protein lysates and subsequent Western blotting to detect the given proteins. Cellular total RNA was also extracted from the indicated cell lines in E for RT-PCR detection of FBXW7.

We were interested in knowing how inhibition of GSK3 blocks rapamycin-induced reduction of cyclin D1, c-Myc and Mcl-1 without interfering with the suppression of mTORC1 signaling and cap-binding by rapamycin. Considering that GSK3 is involved in regulating degradation of these proteins [19–21], we asked whether rapamycin-induced reduction of these proteins is due to enhanced protein degradation. To this end, we first compared the effects of rapamycin on cyclin D1 reduction in the absence and presence of the proteasome inhibitor, MG132. We observed that rapamycin-induced cyclin D1 reduction was prevented by the presence of MG132 in all three tested cell lines (Fig. 5A). Similarly, the presence of MG132 rescued rapamycin-induced reduction of both c-Myc and Mcl-1 (Fig. 4D). Moreover, we determined whether rapamycin affects the stabilities of these proteins. Compared with DMSO control, rapamycin apparently shortened the half-lives of not only cyclin D1, but also c-Myc and Mcl-1 (Fig. 5B), indicating that rapamycin decreases the stabilities of these proteins. Collectively, these data clearly suggest that rapamycin decreases the levels of cyclin D1, c-Myc and Mcl-1 through promoting their degradation.

It is known that the E3 ubiquitin ligase FBX4 is involved in mediating GSK3-dependent degradation of cyclin D1 [18] and FBXW7 mediates GSK3-dependent degradation of both c-Myc and Mcl-1 [19, 20]. Hence, we further determined the impact of knockdown of these E3 ligases on rapamycin-induced reduction of these proteins. As shown in Fig. 5C, knockdown of FBX4 elevated basal levels of cyclin D1 and rescued cyclin D1 reduction induced by rapamycin. When FBXW7 was silenced in both A549 and H460 cells, rapamycin failed to decrease the levels of both c-Myc and Mcl-1 (Fig, 5D). We used reverse transcription-PCR (RT-PCR) to confirm the knockdown efficacies of FBXW7 as presented (Fig. 5D, lower panel). In addition, we also compared the effects of rapamycin on reduction of c-Myc and Mcl-1 between WT and FBXW7-KO HCT116 cell lines and found that rapamycin reduced the levels of c-Myc and Mcl-1 in HCT116 WT cells, but failed to do so in HCT116/FBXW7-KO cells (Fig. 5E). Collectively, these results demonstrate that rapamycin induces FBX4-dependent degradation of cyclin D1 and FBXW7-mediated degradation of c-Myc and Mcl-1, furthering the notion that rapamycin induces GSK3-dependent degradation of cyclin D1, c-Myc and Mcl-1.

### Acute or short-time treatment of cancer cells with rapamycin induces mTORC2 dissociation accompanied with reduction of cyclin D1, c-Myc and Mcl-1

**Figure 6:**
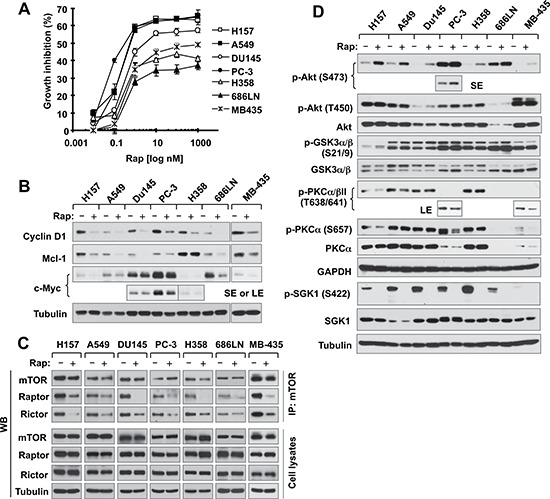
Rapamycin inhibits the growth of various cancer cell lines (A) accompanied with decreasing the levels of cyclin D1, c-Myc and Mcl-1 (B), disrupting the assembly of both mTORC1 and mTORC2 (*C*) and differential effects on the phosohorylation of several AGC kinase proteins (D) **(A)** The indicated cell lines were exposed to various concentrations of rapamycin (Rap) for 3 days. Cell numbers were estimated with the SRB assay. Data, means of four replicated determinations; Bars, ± SDs. **(B and D)** The indicated cell lines were treated with 10 nM rapamycin for 4 h and then cells were harvested for preparation of whole-cell protein lysates and subsequent Western blotting. **(C)** The indicated cell lines were treated with 10 nM rapamycin for 1 h and then harvested for preparation of whole-cell protein lysates followed with immunoprecipitation (IP) using an mTOR antibody and subsequent Western blotting (WB) to detect the indicated proteins. LE, longer exposure; SE, shorter exposure.

We have recently suggested that mTORC2 is responsible for stabilizing cyclin D1 [16]. mTORC2 was initially suggested as a rapamycin-insensitive complex [22, 23] and can be suppressed by prolonged exposure to rapamycin [24]. Despite this, we wanted to determine experimentally whether acute rapamycin treatment affects mTORC2 formation in our tested cancer cell lines. In addition to NSCLC cell lines (H157, A549 and H358), we also included other types of cancer cell lines including prostate cancer (DU145 and PC-3), head and neck cancer (686LN) and breast cancer (MDA-MB-435) cells. These cell lines responded to rapamycin albeit with varied degrees (Fig. 6A). Upon rapamycin treatment for 4 h, we detected cyclin D1 reduction in all 7 tested cell lines, Mcl-1 reduction in 6 of 7 cell lines (except for H358), and c-Myc reduction in 6 of 7 cell lines (except for DU145) (Fig. 6B). These findings indicate that rapamycin induces degradation of these oncogenic proteins across different types of cancer cell lines, implying a common phenomenon.

Following characterization of these cell lines, we treated them with 10 nM rapamycin for 1 h and then detected assembly of mTORCs via mTOR immunoprecipitation (IP) as done previously [24, 25]. To our surprise, we detected reduced levels of not only raptor as expected, but also rictor from IP complexes in all 7 cell lines (Fig. 6C), indicating that rapamycin disrupts the assembly of both mTORC1 and mTORC2 in these cell lines. Following this study, we further examined the effects of acute rapamycin treatment on phosphorylation of Akt, PKCα and SGK1, which are thought to be phosphorylated by mTORC2 at either hydrophobic motif (e.g., Akt S473, PKCα S657 and SGK1 S422) or turn motif (Akt T450 and PKCα T638) [26]. In agreement with our previous observation [27], acute rapamycin treatment increased Akt S473 phosphorylation in every tested cell line; however, we did not see apparent alteration of Akt T450 phosphorylation in any of the cell lines. Interesting, phosphorylation (S21/9) of GSK3, a putative Akt substrate, was only slightly increased by rapamycin in H157 and 686LN cells, but not in other cell lines (Fig. 6D). In contrast to Akt S473 phosphorylation, rapamycin did not increase the phosphorlation of PKCα and SGK1 in any of the tested cell lines. Rather it decreased PKCα T638 phoshorylation in 4 (H157, A549, PC-3 and MB-435) of 7 tested cell lines, PKCα S657 phoshorylation in 3 (H157, PC-3 and MB-435) of 7 tested cell lines and SGK1 S422 phosphorylation in all the tested cell lines (Fig. 6D). Clearly, acute treatment of cancer cells with rapamycin inhibits mTORC2 assembly accompanied with suppression of SGK1 S422 phosphorylation and increase of Akt S473 phosphorylation.

## DISCUSSION

A previous study primarily using GSK3β knockout MEFs has suggested that GSK3β enhances rapamycin-mediated growth inhibition and paclitaxel sensitization [28]. The current study demonstrated that both pharmacological and genetic inhibition of GSK3 compromised or antagonized the growth-inhibitory effects of rapalogs in human NSCLC cells, whereas enforced activation of GSK3 enhanced cell response to rapalogs (Figs. 1–2). Moreover, pharmacological inhibition of GSK3 significantly attenuated the antitumor efficacy of rapalogs *in vivo* against the growth of lung cancer xenografts in nude mice (Fig. 1). Hence this study provides strong preclinical evidence that the presence of GSK3 activity is critical for rapalogs to exert their growth-inhibitory effects and cancer therapeutic efficacies. This notion is further supported by our finding that low basal levels of GSK3 activity in a panel of NSCLC cell lines are significantly correlated with reduced cell responses to rapalogs (Fig. 3).

GSK3 activity is known to be negatively regulated through phosphorylation at Ser 21 (α) and 9 (β) mediated by PI3K/Akt, PKC, p70S6K and RAS/ERK/RSK2 signaling, which are often hyper-activated in a variety of cancer types, [29]. Hence it is reasonable to assume that GSK3 activity should be low in the majority of cancer types; accordingly these cancers will respond poorly to rapalogs. Indeed, our recent pilot study has shown that p-GSK3 is positive in up to 80% of NSCLC specimens, suggesting suppressed GSK3 activity in these tumors [16]. Moreover, rapalogs are known to induce Akt activation while inhibiting mTOR signaling [27, 30]; this will further result in inactivation of GSK3 and confer resistance to rapalogs. Thus, our finding of the critical role of GSK3 in determining cancer response to rapalogs suggests a reasonable scientific base for the poor efficacy of rapalog monotherapy observed in most types of cancers. Accordingly, we suggest that additional enhancement is needed to boost the cancer therapeutic efficacy of rapalogs (e.g., by combination with an agent that may potentially prevent GSK3 inactivation). In this regard, our previous studies have shown that the combination of a rapalog and a PI3K inhibitor (which may activate GSK3 through suppression of Akt) exhibited synergistic anticancer activity [30, 31]. Given that low baseline GSK3 activity is significantly correlated with weak growth-inhibitory effects of rapalogs in cancer cell lines (Fig. 3), further study on GSK3 activity as a possible predictive biomarker for rapalog-based cancer therapy is also warranted.

Several oncogenic proteins, such as cyclin D1, c-Myc and Mcl-1, are known to be regulated by mTORC1 signaling through cap-dependent translation [5, 32]. Although rapamycin reduced the levels of these proteins, this study surprisingly failed to demonstrate that this occurs through mTORC1-mediated suppression of cap-dependent translation, since inhibition of GSK3 prevented reduction of these proteins without rescuing inhibition of phosphorylation of p70S6K, S6 and 4EBP1 and suppression of cap-binding by rapamycin (Fig. 4). Rather, we have demonstrated that rapamycin induces GSK3-dependent degradation of these proteins (Figs. 4 and 5), revealing a novel mechanism by which rapalogs reduce the levels of these oncogenic proteins. Our findings are in fact in agreement with a previous report that rapamycin induces GSK3-dependent degradation of cyclin D1 in human breast cancer cell lines [28]. In addition to these findings, we have further shown that the E3 ubiquitin ligases, FBX4 and FBXW7, mediate rapamycin-induced cyclin D1 degradation and c-Myc and Mcl-1 degradation, respectively (Fig. 5).

Our previous [16] and current studies have shown that both mTOR kinase inhibitors and rapalogs induce GSK3-dependent and FBX4-mediated cyclin D1 degradation. Interestingly, we have further suggested that mTORC2 is actually responsible for stabilizing cyclin D1; hence inhibition of mTORC2 (e.g., with an mTOR kinase inhibitor) triggers its degradation, as we previously suggested [16]. It is unclear whether rapamycin facilitates cyclin D1 degradation through the same mechanism. The challenge is that rapamycin is generally thought to be weak or inactive against the mTORC2. In different systems *in vitro* and *in vivo*, rapamycin has been shown to inhibit mTORC2; however this effect is largely due to chronic or prolonged rapamycin treatment that can eventually disrupt mTORC2 assembly [24, 33–39]. In our study, we observed rapid reduction of cyclin D1, c-Myc and Mcl-1 even at 4 h post rapamycin treatment (Figs. 4C and 6B). Thus, we were interested in knowing whether short-term or acute treatment with a rapalog suppresses mTORC2 under our experimental conditions. Intriguingly, we found that acute treatment with rapamycin (for 1 h) disrupted the assembly of not only mTORC1 (mTOR with raptor), but also mTORC2 (mTOR with rictor) in our tested cell lines (Fig. 6C). When we reviewed the literature carefully, a previous study in fact did show that rictor was dissociated from mTOR at 0.5–2 h post rapamycin in a few cell lines such as PC-3, BJAB and Jurkat [24]. Nonetheless, there are few studies that specifically determine the impact of acute rapamycin treatment on mTORC2 assembly in various types of cancer cell lines. We believe that rapamycin has an acute inhibitory effect against assembly of the mTORC2, at least in some cancer cell lines. Accordingly we suggest that rapalogs, like mTOR kinase inhibitors [16], induce GSK3-dependent degradation of cyclin D1 through inhibition of mTORC2. Elucidation of the mechanism by which mTORC2 inhibition triggers GSK3-dependent degradation of these oncogenic proteins has been ongoing. The outcome of this part of the study may reveal a novel biological function of mTORC2 in regulation of cell growth and survival.

Another interesting observation in this study is that acute rapamycin treatment exerted different effects on the phosphorylation of Akt, PKCα and SGK1, which are known to be mTORC2 substrates [26]. Under our tested conditions, rapamycin clearly reduced p-SGK1 (S422) levels while increasing Akt S473 phosphorylation in every tested cell lines (Fig. 6D). It appears that disruption of the mTORC2 assembly is associated well with suppression of SGK1 S422 phosphorylation. Whether SGK1 S422 phosphorylation serves as a better readout of mTORC2 activity than Akt S473 phosphorylation needs further investigation.

**Figure 7:**
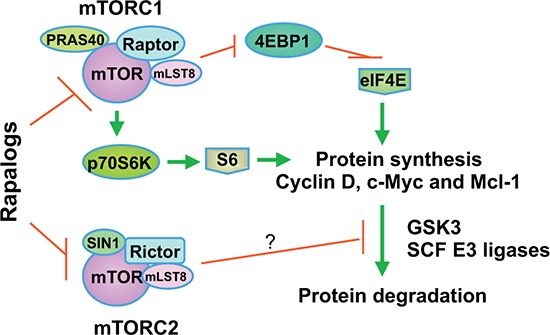
A schema highlighting key findings in this study In addition to positive regulation of translation or synthesis of proteins such as cyclin D1, c-Myc and Mcl-1 by mTORC1 through suppression of 4EBP1 and activation of p70S6K, our previous and current findings suggest that mTORC2 also stabilize these proteins through inhibiting their degradation. We suggest that rapalogs primarily decrease the levels of cyclin D1, Mcl-1 and c-Myc by promoting GSK3-dependent degradation of these proteins via inhibition of mTORC2.

In summary, this study has demonstrated the novel activity of rapamycin in inducing GSK3-dependent degradation of several oncogenic proteins including cyclin D1, c-Myc and Mcl-1. This effect is likely the consequence of mTORC2 inhibition and may at least in part accounts for the growth-inhibitory effects and anticancer activity of rapalogs (Fig. 7). Thus our findings highlight a novel mechanism by which rapalogs exert their biological function and anticancer activity.

## MATERIALS AND METHODS

### Reagents

Rapamycin and CHIR99021 were purchased from LC laboratories (Woburn, MA). RAD001 was supplied by Novartis Pharmaceuticals Corporation (East Hanover, NJ). SB216763, MG132 and cyclohexemide (CHX) were purchased from Sigma Chemical Co. (St. Louis, MO). Cyclin D1, Mcl-1, c-Myc, p-GSK3α/β(S21/9), p-AKT (S473), p-Akt (T450), p-PKCα/βII (T638/641), mTOR, raptor, AKT, p-S6 (S235/236), and S6 antibodies were purchased from Cell Signaling Technology, Inc. (Danvers, MA). GSK3α/β, PKCα and SGK1 antibodies were purchased from EMD Millipore (Billerica, MA). mTOR (FRAP; N-19), p-PKCα (S657) and p-SGK1 (S422) antibodies were purchased from Santa Cruz Biotechnology, Inc. (Santa Cruz, CA). Rictor (BL2178) antibody was purchased from Bethyl Laboratories, Inc. (Montgomery, TX). Both polyclonal and monoclonal actin antibodies were purchased from Sigma Chemical Co. Myc-tagged constitutively active form of GSK3β (GSK3βCA) [40] was provided by Dr. B. P. Zhou (The University of Kentucky, College of Medicine, Lexington, Kentucky).

### Cell lines and cell culture

NSCLC cell lines used in this study were described in our previous work [31]. WT, GSK3α-KO and GSK3β-KO MEFs were generously provided by Dr. J. Woodgett (Samuel Lunenfeld Research Institute, Mount Sinai Hospital, Toronto, Canada). HCT116/WT and HCT116/FBXW7-KO cell lines were kindly provided by Dr. B. Vogelstein (Johns Hopkins University School of Medicine, Baltimore, MA). A549-pLKO.1, A549-shFBX4-#1 and A549-shFBX4-#5 cells were described previously [16]. Except for H157 and A549 cells, which were authenticated by Genetica DNA Laboratories, Inc. (Cincinnati, OH) through analyzing short tandem repeat DNA profile, other cell lines have not been authenticated. These cell lines were cultured in RPMI 1640 or DMEM medium containing 5% fetal bovine serum at 37°C in a humidified atmosphere of 5% CO_2_ and 95% air

### Cell growth assay

Cells were seeded in 96-well cell culture plates and treated the next day with the given agents. The cell number was determined using sulforhodamine B (SRB) assay as described previously [41]. CI for drug interaction (e.g., synergy) was calculated using the CompuSyn software (ComboSyn, Inc.; Paramus, NJ).

### Cell cycle analysis

Cells were harvested after a given treatment and stained with propidium iodide for cell cycle analysis as described previously [42, 43].

### Colony formation assay

The effects of the given drugs on colony formation on plates were measured as previously described [44].

### Western blot analysis

Preparation of whole-cell protein lysates and performance of the Western blot analysis were the same as described previously [45, 46].

### Gene knockdown by siRNA

The non-silencing control, GSK-3α, GSK-3β and GSK3α/β siRNAs were described previously [16]. FBXW7 siRNA was the same as described previously [47]. Transfection of these siRNA duplexes was conducted in 6-well plates using the HiPerFect transfection reagent (Qiagen) following the manufacturer's manual. The knockdown effects of FBXW7 siRNA were evaluated with RT-PCR as described previously [47] due to lack of a specific antibody for Western blotting.

### m^7^GTP pull-down for analysis of eIF4F complex

eIF4F complex in cell extracts was detected using affinity chromatography m^7^GTP-sepharose as described previously [48].

### Detection of mTORCs

mTORCs were detected by immunoprecipitating mTOR with mTOR (FRAP; N-19) antibody followed with Western blotting to detect raptor and rictor according to the same procedure described previously [22, 25].

### Lung cancer xenografts and treatments

Lung cancer xenograft experiments were approved by the Institutional Animal Care and Use Committee (IACUC) of Emory University and conducted as previously described [16]. Five- to 6-week old (about 20 g of body weight) female athymic (nu/nu) mice were ordered from Harlan (Indianapolis, IN). Mice (6/group) received the following treatments: vehicle control, RAD001 (1 mg/kg/day, o.g.), SB216763 in DMSO (5 mg/kg/day; i.p.), and the combination of RAD001 and SB216763. This study was done in conjunction with our previous INK128 and SB216763 combination experiment as described in our previous study [16] and thus the vehicle and SB216763 groups were shared to minimally use mice.

### Statistical analysis

The statistical significance of differences between two groups was analyzed with two-sided unpaired Student's *t* tests when the variances were equal or with Welch's corrected *t* test when the variances were not equal by use of Graphpad InStat 3 software (GraphPad Software, San Diego, CA). Data were examined as suggested by the same software to verify that the assumptions for use of the *t* tests held. Results were considered to be statistically significant at *P* < 0.05.

## SUPPLEMENTARY FIGURES


