# GSK3 is required for rapalogs to induce degradation of some oncogenic proteins and to suppress cancer cell growth

**DOI:** 10.18632/oncotarget.27752

**Published:** 2021-02-02

**Authors:** Junghui Koo, Xuerong Wang, Taofeek K. Owonikoko, Suresh S. Ramalingam, Fadlo R. Khuri, Shi-Yong Sun

**Affiliations:** ^1^ Department of Hematology and Medical Oncology, Emory University School of Medicine and Winship Cancer Institute, Atlanta, GA, USA; ^2^ Department of Pharmacology, Nanjing Medical University, Nanjing, Jiangsu, China

**This article has been corrected:** In Figure 4A, the image of 4EBP1 in A549 at 6 h is an accidental duplicate of the image of 4EBP1 in H460 at 6 h. In Figure 6C, the image for mTOR for ‘cell lysates’ of MB-435 is an accidental duplicate of the image for mTor for ‘IP: mTor’ of MB-435. Both corrected figures, using the proper images obtained from the original data, are shown below. The authors declare that these corrections do not change the results or conclusions of this paper.

Original article: Oncotarget. 2015; 6:8974–8987. 8974-8987. https://doi.org/10.18632/oncotarget.3291

**Figure 4:**
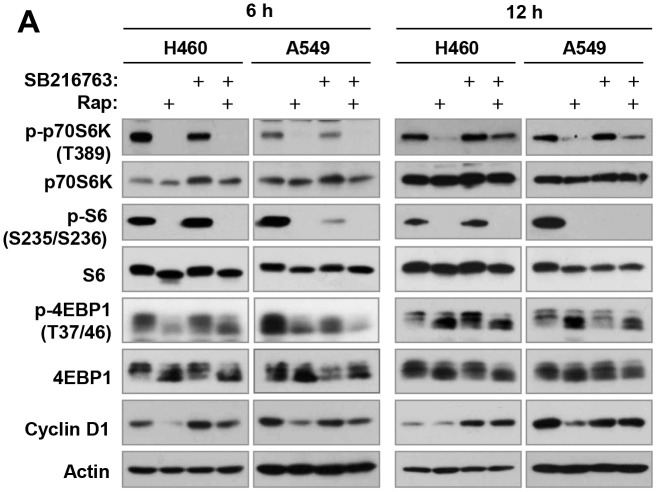
Inhibition of GSK3 with SB216763 or siRNA rescues rapamycin-induced reduction of cyclin D1, c-Myc and Mcl-1 (A, C-F) without blocking rapamycin-mediated suppressive effects on mTORC1 signaling (A) and on cap binding (B). (**A** and **B**), The indicated cell lines were treated with DMSO, 10 nM rapamycin (Rap), 5 μM SB216763, or rapamycin plus SB216763 for 6 h or 12 h. (**C**), The indicated cell lines were exposed to different concentrations of rapamycin for 4 or 8 h. (**D**), The indicated cell lines were treated with DMSO, 10 nM rapamycin, 5 μM SB216763, 10 μM MG132, rapamycin plus SB216763, or rapamycin plus MG132 for 6 h. (**E**), The indicated cell lines were treated with DMSO, 10 nM rapamycin, 10 μM CHIR99021, or rapamycin plus CHIR99021 for 6 h. (**F**), A549 cells were transfected with the given siRNAs and after 48 h were exposed to 10 nM rapamycin for 6 h. After the aforementioned treatments, the cells were then harvested for preparation of whole-cell protein lysates and subsequent Western blotting. Moreover, lysates from H460 cells (B) were also used to the m7GTP pull-down and subsequent detection of the given proteins by Western blot analysis (B).

**Figure 6:**
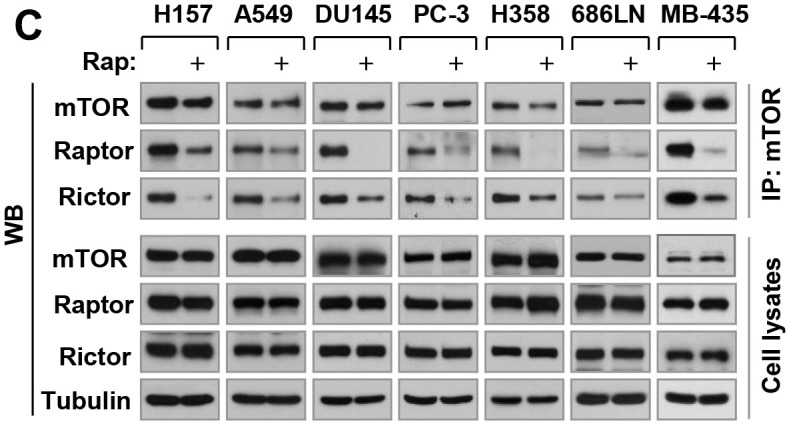
Rapamycin inhibits the growth of various cancer cell lines (A) accompanied with decreasing the levels of cyclin D1, c-Myc and Mcl-1 (B), disrupting the assembly of both mTORC1 and mTORC2 (C) and differential effects on the phosohorylation of several AGC kinase proteins (D). (**A**) The indicated cell lines were exposed to various concentrations of rapamycin (Rap) for 3 days. Cell numbers were estimated with the SRB assay. Data, means of four replicated determinations; Bars, ± SDs. (**B** and **D**) The indicated cell lines were treated with 10 nM rapamycin for 4 h and then cells were harvested for preparation of whole-cell protein lysates and subsequent Western blotting. (**C**) The indicated cell lines were treated with 10 nM rapamycin for 1 h and then harvested for preparation of whole-cell protein lysates followed with immunoprecipitation (IP) using an mTOR antibody and subsequent Western blotting (WB) to detect the indicated proteins. LE, longer exposure; SE, shorter exposure.

