# Supplementary material for: GSK3 is required for rapalogs to induce degradation of some oncogenic proteins and to suppress cancer cell growth
Source: Oncotarget. 2015 Mar 12;6(11):8974–87. doi: 10.18632/oncotarget.3291 (PMC4496196; doi:10.18632/oncotarget.3291)
Supplement: Supplementary file 1 [file oncotarget-06-8974-s001.pdf]

SUPPLEMENTARY FIGURES

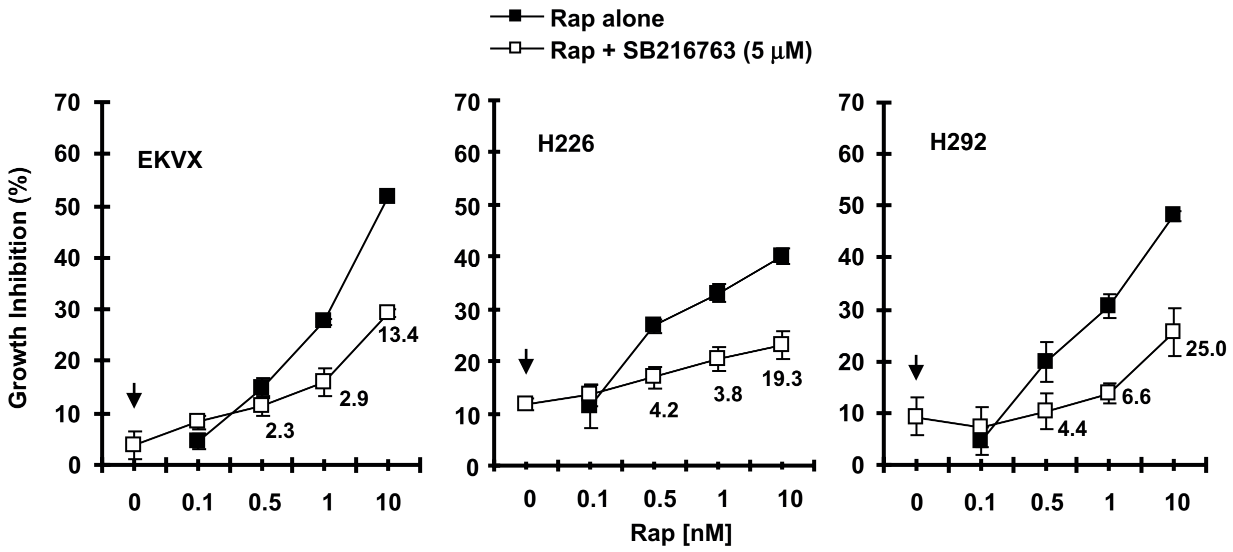

Supplementary Figure S1: Inhibition of GSK3 with SB216763 antagonizes rapalycin's growth-inhibitory effects in other lung cancer cell lines. The given lung cancer cell lines were plated on 96-well cell culture plates and treated next day with the indicated concentrations of rapamycin (Rap) alone, SB216763 (SB) alone (as indicated by arrow inside the graphs) or their combination. After 3 days, cell numbers were estimated using the SRB assay and CIs were calculated with CompuSyn software and labeled inside the graphs. Data, means of four replicated determinations; Bars, ± SDs.
